# Neotropical bats that co-habit with humans function as dead-end hosts for dengue virus

**DOI:** 10.1371/journal.pntd.0005537

**Published:** 2017-05-18

**Authors:** Amanda Vicente-Santos, Andres Moreira-Soto, Claudio Soto-Garita, Luis Guillermo Chaverri, Andrea Chaves, Jan Felix Drexler, Juan Alberto Morales, Alejandro Alfaro-Alarcón, Bernal Rodríguez-Herrera, Eugenia Corrales-Aguilar

**Affiliations:** 1 Virology-CIET (Research Center for Tropical Diseases), Microbiology, University of Costa Rica, San José, Costa Rica; 2 Biology, University of Costa Rica, San José, Costa Rica; 3 Institute of Virology, University of Bonn Medical Centre, Bonn, Germany; 4 Exact and Natural Sciences School, National Distance Education University, San José, Costa Rica; 5 German Centre for Infection Research, Bonn-Cologne, Germany; 6 Department of Pathology, School of Veterinary Medicine, National University, Heredia, Costa Rica; University of Washington, UNITED STATES

## Abstract

Several studies have shown Dengue Virus (DENV) nucleic acids and/or antibodies present in Neotropical wildlife including bats, suggesting that some bat species may be susceptible to DENV infection. Here we aim to elucidate the role of house-roosting bats in the DENV transmission cycle. Bats were sampled in households located in high and low dengue incidence regions during rainy and dry seasons in Costa Rica. We captured 318 bats from 12 different species in 29 households. Necropsies were performed in 205 bats to analyze virus presence in heart, lung, spleen, liver, intestine, kidney, and brain tissue. Histopathology studies from all organs showed no significant findings of disease or infection. Sera were analyzed by PRNT_90_ for a seroprevalence of 21.2% (51/241), and by PCR for 8.8% (28/318) positive bats for DENV RNA. From these 28 bats, 11 intestine samples were analyzed by RT-PCR. Two intestines were DENV RNA positive for the same dengue serotype detected in blood. Viral isolation from all positive organs or blood was unsuccessful. Additionally, viral load analyses in positive blood samples by qRT-PCR showed virus concentrations under the minimal dose required for mosquito infection. Simultaneously, 651 mosquitoes were collected using EVS-CO_2_ traps and analyzed for DENV and feeding preferences (bat cytochrome b). Only three mosquitoes were found DENV positive and none was positive for bat cytochrome b. Our results suggest an accidental presence of DENV in bats probably caused from oral ingestion of infected mosquitoes. Phylogenetic analyses suggest also a spillover event from humans to bats. Therefore, we conclude that bats in these urban environments do not sustain DENV amplification, they do not have a role as reservoirs, but function as epidemiological dead end hosts for this virus.

## Author summary

Dengue is the most important human vector-borne disease. Several studies have shown DENV presence in mammalian wildlife such as bats, thus considering them putative reservoirs or hosts. We aimed to elucidate if bats that cohabit in houses in close proximity with humans may be involved in a dengue transmission cycle. We sampled bats in low and high dengue incidence areas during the dry (low mosquito abundance) and wet (high mosquito abundance) seasons. We analyzed blood and several organs. As previously reported, we found DENV nucleic acid and neutralizing antibodies in a small percentage of blood samples, but virus detection in all organs was negative. We were able to show that dengue found in all positive samples was in low concentration and thus virus isolation was unsuccessful. We found positive intestine samples which may suggest infection through DENV-positive mosquito ingestion. Furthermore, mosquitoes sampled in close vicinity of bats’ roosting place were not feeding on these mammals. Virus sequence analysis from bats and humans show a spillover effect from humans to bats. Taken together, our results indicate that bats do not sustain sufficient virus amplification in order to function as reservoirs and exclude them as players in the dengue virus transmission cycle.

## Introduction

Dengue is the most important arthropod-borne viral infection of humans, it is established in the tropics worldwide, and its geographical expansion is expected to increase due to factors such as modern dynamics of climate change, globalization, travel, trade, poverty, unplanned urbanization, and viral evolution [1]. The World Health Organization (WHO) estimates that 2.5 billion people are at risk of infection, with 50–100 million infections per year [2].

Currently, two distinct and independent DENV transmission cycles occur: (i) Endemic DENV circulates among humans functioning as reservoirs and amplification hosts, transmitted mainly by peridomestic *Ae*. *aegypti* and *Ae*. *albopictus* mosquitoes; and (ii) Sylvatic DENV that circulates among non-human primate reservoir hosts transmitted by several different *Aedes* mosquitoes found in forested habitats of West Africa and Southeast Asia [3]. In the New World, dengue infection is thought to be absent in wildlife, as the endemic transmission cycle was introduced by its vectors *Ae*. *aegypti* and *Ae*. *albopictus* to the Neotropics [3]. However, several studies have documented the presence of DENV RNA and/or antibodies against DENV in mammalian wildlife in the Neotropics [4–10]. However which mosquito species might function as transmission vectors in the forest/jungle are yet unknown, since *Ae*. *aegypti* is predominantly associated with urban sites [8]. Antibodies against DENV were found in bat sera by plaque reduction neutralization test (PRNT) in a study conducted in Costa Rica and Ecuador [4]. They observed that *Ae*. *aegypti* fed on bats in controlled laboratory conditions, suggesting that these mammals could play a role in the virus cycle as possible reservoirs. In French Guiana, another study determined the presence of neutralizing antibodies against flavivirus in wild mammals (armadillos, porcupines, opossums, agoutis, and wild goats) of pristine forests [6]. The authors proposed that wild animals can be exposed to DENV thus functioning as temporary reservoirs. A study conducted in high dengue incidence areas from the Mexican Pacific coast found four bats species positive for DENV-2 using different techniques [7], suggesting their role in maintaining DENV in nature. Another study in French Guiana detected all four serotypes in liver samples and/or serum of 92 wild mammals (bats, rodents and marsupials) [5,8]. By short sequence analysis of the C/prM region they determined that DENV-1, DENV-3, and DENV-4 were different from those DENV co-circulating in humans in the same geographical area. Furthermore, the analysis of DENV-2 short sequences found in mammals showed that some wild DENV strains seemed to diverge from concurrent human strains, though others were identical. They indicated that neotropical mammals living in peri-urban areas may encounter DENV strains circulating in humans, and under pressure from a strong epidemic event, urban strains could be introduced into the forest and infect wildlife fauna [5,8]. The authors emphasize that it is important to consider wildlife not only as potential reservoirs for DENV, but also as potential hosts sensitive to infections by human pathogens [5]. They suggested that these species can be an epidemic dead end or play a role in maintaining the virus during epidemic inter-periods. Another serological survey conducted in Mexico found flavivirus-specific antibodies in 19% of bats tested [10]. Here the PRNT titers against DENV were higher than for other flaviviruses, however since all neutralization titers were considered low this prompted them to the conclusion that bats may have been infected with another flavivirus not included in the analysis [10]. Additionally, DENV-2 was found in spleens of 6 bats captured from anthropogenically changed and unaltered landscapes in southern Mexico [9]. They do not report any effect of anthropogenic disturbance on the occurrence of DENV.

Two recent studies have tested the susceptibility of bats for DENV infection in laboratory controlled conditions. Perea-Martínez *et al*. [11] showed that after intraperitoneally inoculation, DENV-2 replicates poorly on *Artibeus intermedius* bats suggesting that they are not suitable hosts for this virus. In addition, another study inoculated *Artibeus jamaicensis* bats with DENV-1 and DENV-4 using different routes: subcutaneously, intraperitoneally, and bitten by infected *Ae*. *aegypti*. They detected DENV RNA (6/22) in spleen and NS1 (8/22) in serum in some cases, though in low concentrations and in a non-reproducible manner [12]. The authors concluded that bats are thus incapable of sustaining DENV replication and are unlikely to act as reservoirs for this virus.

More than 200 viruses from 27 different families, including *Flaviviridae*, have been isolated or detected in bats [13]. However, whether bats are simply incidental virus hosts or serve as competent reservoirs able to transmit these viruses to other vertebrates are open questions that must be carefully addressed [13–20]. Bats are extremely important components of biodiversity: their role in forest regeneration and insect pest control is well known [21,22]. The privileged geographical location of Costa Rica favors a rich bat biodiversity, representing this land’s largest order of mammals [23]. In Costa Rica, 10% of houses (mainly ceilings and rooftops) are colonized by bats, and community reports of bats colonization in neighboring buildings such as schools and churches are common [24]. This indicates a close proximity of humans with these wild animals in this country.

The present study aims to elucidate a putative cycle of dengue viral transmission involving humans, mosquitoes and bats in places where they could interact: household environments. Different angles of this possible role were comprehensively achieved by molecular and serological diagnosis, histopathological analyses, phylogenetic relationship of the viral agent, and integration with anthropogenic and ecological factors.

## Methods

### Ethics statement

All bat specimens were collected following the recommendations of the Institutional Committee of Care and Use of Animals of the University of Costa Rica (IACUC) (CICUA-36-13) according to national guidelines for animal caring described in the Costa Rica National Law for Animal Welfare 7451. After signing an informed consent approved by the University of Costa Rica’s Scientific Ethics Committee (CEC) according to the principles expressed in the Declaration of Helsinki (CEC VI-3970-2013), a blood sample from humans was taken for IgG ELISA analysis.

### Bats, mosquitoes, and humans sampling

Sampling was performed during the rainy (more than 250 mm rainfall/month) and dry (less than 100 mm rainfall/month) [25] seasons of 2013 and 2014 in three different locations with dengue low and high incidence according to the reported dengue cases by the National Ministry of Health [26] (S1 Fig). The first site, La Virgen from Sarapiquí (10°24’20”N, 84°8’3”W), is a rural area surrounded by rainforest and agricultural fields with high incidence of dengue. The second site, Nicoya (10°9’42”N, 85°26’48”W) is a peri-urban area located between pastures and dry forest with dengue high incidence. The third site was the Central Valley (9°55’42”N, 84°8’35”W), where the Great Metropolitan Urban area is found and has low incidence of dengue. At each site, at least 5 houses where humans and bats cohabit were located using a snowball sampling strategy and sampled during both seasons. Bats were captured with mist nets positioned at their root’s exit or directly by hand from the ceiling. Captured animals were taxonomically identified [27]. Age, sex, and reproductive status were also determined. Five bats per household were euthanized by intra-muscular anesthesia overdose (ketamine 10 mg/kg + xylazine 1 mg/kg). Complete gross necropsies were performed from aseptically collected heart, lung, liver, spleen, kidney, intestine, and brain. A segment of each organ was preserved at -80°C with 200 μl of RNAlater Stabilization Solution (Life Technologies, Thermo Fisher Scientific Inc.). Another segment was preserved in 10% neutral buffered formalin for histopathology analyses. Remaining bats captured from each household were released after blood sampling by puncture of a branchial vein. Coagula and plasma were stored at -80°C for later analysis.

In parallel, four EVS-CO_2_ Traps (BioQuip Products, CA, USA) were placed inside and outside of each sampled household during 16–20 hours. Collected mosquitoes were frozen in dry ice, identified to species or genus [28], and stored at -80°C for later PCR analysis. Mosquito breeding sites near or inside the households were also located for larvae collection.

A survey was conducted in each household to determine previously diagnosed or suspected human dengue infections, social and economic aspects, and their interaction with bats.

### Molecular methods, histopathology, viral isolation and microneutralization assays of collected bat samples

Viral RNA was extracted from blood and from a pool of collected organs using TRIzol Reagent (Invitrogen, Carlsbad CA, USA) according to the manufacturer’s instructions. cDNA was synthesized using RevertAid H Minus Kit (Fermentas, ThermoFisher Scientific, USA) with random hexamers or the D1 forward primer [29], according to the manufacturer’s instructions. All primers used and their references are found in the S1 Table. A seminested-PCR was performed following the protocol previously described [29]. Briefly, PCR was performed using cDNA and the D1 and D2 primers, amplifying a fragment of 511 bp from the capsid and premembrane (C/prM) genes. The second amplification was performed using a dilution of the first PCR product, and the primers D1 and TS1-TS2-TS3-TS4, generating PCR products of different sizes for each serotype (482bp for DENV-1, 119bp for DENV-2, 290bp for DENV-3, and 392bp for DENV-4). Positive controls (DENV-1 Angola (D1/AO/XX/1988), DENV-2 Jamaica (D2/JM/1409/1983), DENV-3 Nicaragua (D3/NI/30-94/1994), DENV-4 Dominica (D4/DM/ 814669/1981)) and negative control (water) were present in each run for serotype confirmation and to rule out cross-contamination. If whole blood was found positive for DENV RNA, single organ PCR back analyses were then performed in heart, lung, liver, spleen, kidney, brain, and intestine (when collected) separately.

Positive blood samples for DENV RNA were also analyzed by a quantitative Real Time PCR (qRT-PCR) assay as described elsewhere [30]. A StepOne RT-PCR System (Applied Biosystems, ThermoFisher Scientific) with the SuperScript III OneStep RT-PCR System, with Platinum Taq kit (Invitrogen, ThermoFisher Scientific), and the primers and probe (DEN IVT) designed by Drosten et al. [30] were used. Quantification was performed using a standard curve generated with log_10_ probe dilutions.

Tissue samples fixed in 10% neutral buffered formalin were embedded in paraffin, sectioned at 3 μm, and stained following standard procedures [31]. Complete histopathological examination of tissues was done.

Viral isolation was attempted from DENV PCR positive blood samples. 48-well flat-bottomed cell culture plates were seeded with 2.5 x 10E5 C6/36 (*Aedes albopictus* cell line ATCC Number: CRL-1660) in RPMI 1640 medium with GlutaMAX-I (Gibco, BRL) supplemented with 2% fetal bovine serum (Gibco, BRL), penicillin (100 units/ml) and streptomycin (100 μg/ml) (Sigma-Aldrich, USA). Coagula with plasma rest were washed with 50 μl sterile PBS, centrifuged and 20 μl of the supernatant was inoculated in each duplicate well. Cells were incubated at 28°C in a 5% CO_2_ atmosphere during 24 hours for virus adsorption, then medium was changed and further incubated during 15 days. Afterwards, cells were passaged into 25cm^2^ cell culture flasks, and incubated for 30 more days. Cells were observed daily for appearance of viral cytopathic effect (CPE), and analyzed periodically for DENV RNA by PCR.

Bat sera were analyzed in microneutralization assays performed in 96-well, flat-bottomed tissue culture plates with Vero cells (ATCC Number: CCL-81). Serum samples were heat-inactivated at 57°C for 30 min, and diluted 1:10 in MEM 2% FCS. Only one dilution was tested due to limited sera amount from some bat species. 30 μl of virus inoculum with different PFU amounts (DENV-1 Angola (D1/AO/XX/1988), 100 PFU; DENV-2 Jamaica (D2/JM/1409/1983), 150 PFU; DENV-3 Nicaragua (D3/NI/30-94/1994), 150 PFU; DENV-4 Dominica (D4/DM/ 814669/1981), 200 PFU) were mixed with an equal volume of serum dilution and incubated 1 h at 37°C. Then, 50 μl of the serum-virus mixture was placed into Vero cells and incubated 90 min at 37°C. After adsorption, the serum-virus inoculum was removed and 100 μl of 1.5% carboxymethylcellulose (CMC) (Sigma-Aldrich, USA) overlay medium were added. Plates were incubated at 37°C in an atmosphere of 5% CO_2_ for 72 hours for DENV-1, DENV-2, and DENV-3, and for 48 hours for DENV-4. As assay controls, a positive human serum pool (sera previously determined to possess high ELISA titers against all four DENV serotypes), negative human serum, and mock cell controls were included. After the incubation period, the CMC overlay medium was removed, cells were fixed with methanol at -20°C and stained with the monoclonal Dengue virus 1, 2, 3 & 4 antibody [D1-11(3)] (GeneTex, CA, USA) for DENV-1, DEN-2 and DENV-3 and with the 4G2 antibody (Hennessey Research, Inc.) for DENV-4. Foci were counted visually. A 90% reduction of foci number at the 1:20 serum dilution was considered positive.

### DENV RNA presence and blood meal preferences from collected mosquitoes

Mosquitoes were sorted by date, house, trap and species [28]. Female mosquitoes were dissected in heads and abdomens. Forceps and surgical blades used were sterilized in ethanol, flamed and immersed in DNA Away (Molecular Bioproducts Inc., CA, USA) between dissections to avoid cross-contamination. Pools of 25 or fewer individuals were macerated and homogenized in 200 μl of RNAlater Stabilization Solution (Life Technologies, Thermo Fisher Scientific Inc.). Viral RNA extraction, cDNA retro-transcription and DENV PCR were performed as described above.

For blood meal preference analysis of mosquitoes, DNA was extracted from gut pools using NucleoSpin Tissue (Macherey-Nagel, Germany) according to the manufacturer’s instructions. Gene segments were amplified with two sets of primers that amplify overlapping regions of mitochondrial cytochrome oxidase subunit I (COI), COI_short and COI_long, and one primer set for cytochrome b (Cyt *b*) as described in Townzen et al. [32]. PCR products were purified using Exonuclease I and Thermo Scientific FastAP Thermosensitive Alkaline Phosphatase (Thermo Fisher Scientific Inc.) following the manufacturer’s protocol. Both strands of the amplicons were sequenced by Macrogen Inc. (Seoul, South Korea). Each obtained sequence was compared with entries in GenBank using the nucleotide basic local alignment search tool (BLASTn) (http://www.ncbi.nlm.nih.gov/).

### DENV IgG detection in human serum samples

DENV-specific IgG titers in human serum samples were determined by a sandwich-like enzyme-linked immunosorbent assay (ELISA) system (AccuDiag Dengue IgG ELISA kit; Diagnostic Automation, Inc., Calabasas, CA). Analysis of the samples and the interpretation of positive or negative ELISA reactions were made according to the manufacturer's instructions.

### Phylogenetic analysis of obtained DENV sequences

After dengue serotype identification of positive samples, cDNA segments were amplified by two different methodologies for phylogenetic analysis. First, cDNA segments between 2,474 and 2,577 nucleotides encompassing the prM and E genes of DENV were amplified by PCR using the consensus primer D1 and serotype-specific reverse primers as in Díaz et al. [33] with modifications for DENV-3, since the annealing temperatures were changed to 51°C and 60°C. Additionally, cDNA segments between 424 bp and 461 bp including the C/prM region or DENV were amplified using the first PCR products from the [29] seminested-PCR for another seminested-PCR amplification using D1 and one of serotype-specific reverse primers as described elsewhere [5]. PCR products were purified using Exonuclease I and Thermo Scientific FastAP Thermosensitive Alkaline Phosphatase (Thermo Fisher Scientific Inc.) following the manufacturer’s protocol. Both strands of the amplicons were sequenced in Macrogen Inc. (Seoul, South Korea), using either the primers from Díaz et al. [33] or the amplification primers from de Thoisy et al. [5], respectively. Obtained sequences from bats, mosquitoes, and from already published dengue isolates in Costa Rica [34] were aligned with previously published sequences of dengue virus in GenBank databases (S4 and S5 Tables) using MEGA v6.0 software. Alignments were checked manually. The identification of the best nucleotide substitution model and the construction of phylogenetic trees using the maximum likelihood statistical method were performed using MEGA v6.0 (www.megasoftware.net). The robustness of the resulting tree was established by bootstrap analysis with 1,000 replications.

### Statistical analysis

For the DENV RNA results, factors such as gender, sampling site (Sarapiquí, Nicoya or Central Valley), and season (dry or rainy) were subjected to the chi-squared test (χ^2^). While for the seropositivity, factors such as gender, age (juvenile or adult), reproductive status (inactive, pregnancy, lactation), and sampling site were tested. A GLMM Binomial was performed in order to test correlation between human IgG anti-DENV presence and bat DENV RNA presence. Mosquito abundance was tested by sampling site and season with Kruskal-Wallis. Analyses of the data were done using R v3.2.1 software [35].

## Results

### A limited number of bats roosting in households have DENV RNA or antibodies against DENV in blood

**Fig 1:**
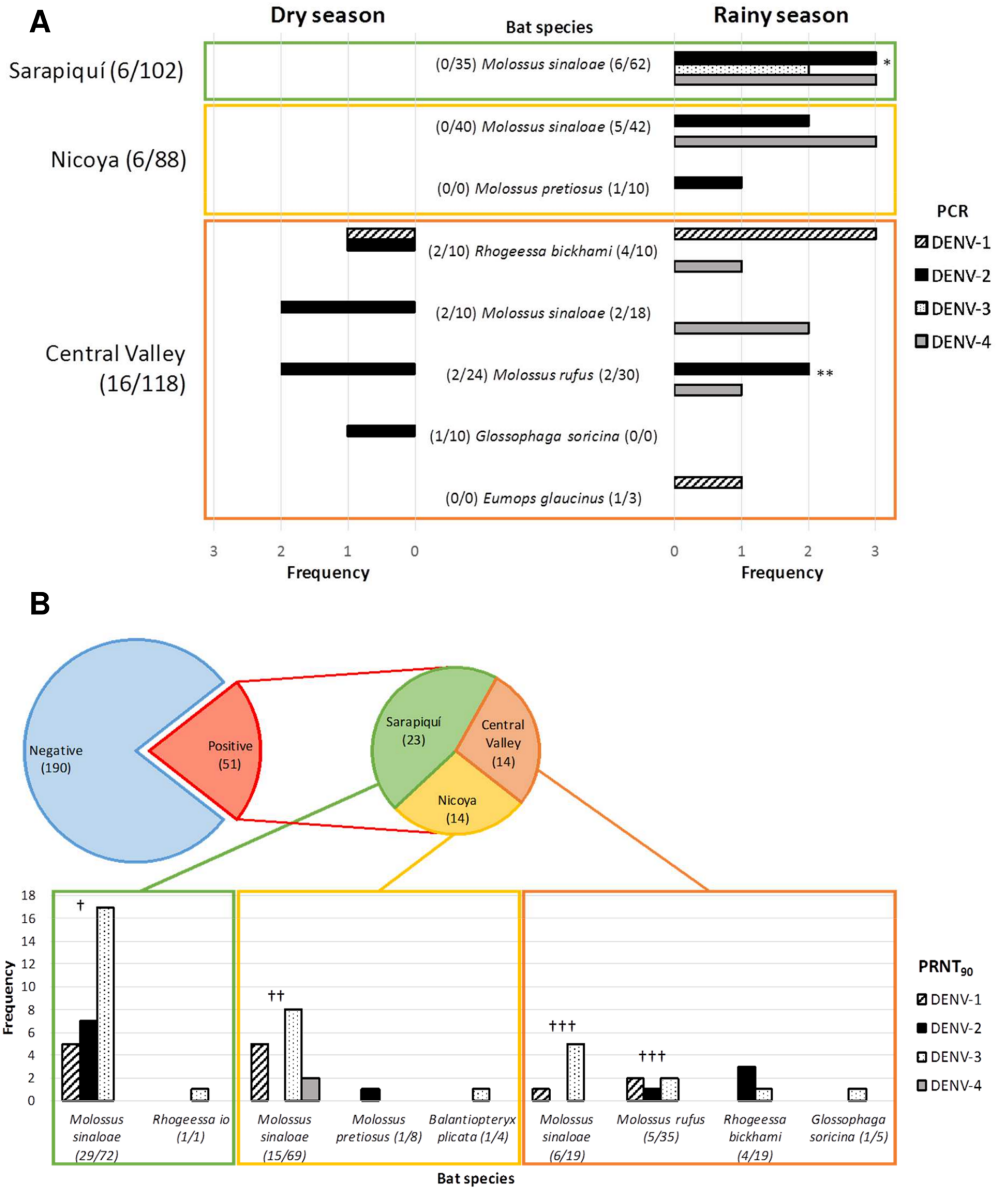
Detection of DENV RNA and antibodies against DENV in the captured bats in the 3 sites of study in Costa Rica (Nicoya, Sarapiquí and Central Valley, S1 Fig) during the dry and rainy season, 2013–2014. (**A)** Prevalence of DENV RNA obtained by PCR from each serotype of DENV in positive bat species. *Two individuals with joint detection of DENV-2 and DENV-4. **One individual with joint detection of DENV-2 and DENV-3. (**B)** Seroprevalence against each dengue serotype obtained from serum diluted 1:20 by PRNT_90_ from positive bat species. Twelve individuals presenting antibodies against more than one serotype: † DENV-1 & DENV-3 (3) and DENV-2 & DENV-3 (4); † † DENV-1 & DENV-3 (2) and DENV-1 & DENV-4 (1); † † † DENV-1 & DENV-3 (2).

A total of 318 bats from twelve different species were captured: *Balantiopteryx plicata* (5), *Eptesicus fuscus* (3), *Eumops glaucinus* (3), *Glossophaga soricina* (10), *Molossus pretiosus* (10), *Molossus rufus* (54), *Molossus sinaloae* (207), *Myotis elegans* (1), *Myotis nigricans* (3), *Rhogeessa io* (1), *Rhogeessa bickhami* (20), and *Uroderma convexum* (1). Part (n = 205) was collected and euthanized, and blood samples was taken from the rest. The blood positive samples (8.8%, 28/318; Fig 1A, S2 Table) were confirmed by sequencing. We found DENV RNA in the species *E*. *glaucinus* (1/3), *G*. *soricina* (1/10), *M*. *pretiosus* (1/10), *M*. *rufus* (5/54), *M*. *sinaloae* (17/207), and *R*. *bickhami* (6/20). RNA of DENV-1 was present in 17.8% (5/28), DENV-2 in 50% (14/28), DENV-3 in 7% (2/28), and DENV-4 in 35.7% (10/28) of the positive blood samples. Interestingly, we found two individuals exposing double RNA presence with DENV-2 and DENV-4 (*M*. *sinaloae*), and one with DENV-2 and DENV-3 (*M*. *rufus*).

Considering geographic samples, at Sarapiquí 7.8% of the bats sampled (8/102) were DENV-2 (3/8), DENV-3 (2/8), and DEN-4 (3/8) positive. At Nicoya, 6.1% of the bats sampled (6/98) were DENV-2 (3/6) and DENV-4 (3/6) positive. Finally, at the Central Valley, 14.4% of the bats sampled (17/118) had DENV-1 (5/17), DENV-2 (8/17) and DENV-4 (4/17) RNA in blood samples. Surprisingly, the Central Valley was the only site where DENV RNA was detected during the dry season (7 positive bats) even though it is considered to be a low dengue incidence region. We found no differences of DENV positivity between gender (χ^2^ = 0.09, df = 1, p = 0.76), sampling site (χ^2^ = 3.40, df = 2, p = 0.18) or season (χ^2^ = 1.87, df = 1, p = 0.17).

To detect putative viral replication sites, bat organ pools were analyzed for DENV RNA presence. We did not detect any DENV RNA in any organ pool. To exclude possible dilution of the DENV RNA in the pool hence the negative results, we analyzed individually then each organ from the 28 DENV RNA in blood positive bats. Interestingly, nor heart, lung, liver, spleen, kidney, or brain were found positive for viral RNA. No pathological lesions in the tissue organs sampled were observed. Additionally, we had collected some intestines (11) from the 28 DENV RNA in blood positive bats. Surprisingly, after DENV RNA analysis two individuals had positive DENV RNA PCR results, coinciding with the previously detected serotype in their respective blood. Nevertheless, we are not able to exclude false positives from intestinal content rests which may include DENV-positive mosquitoes, since both were insectivorous bats (*M*. *sinaloae*).

No dengue virus was successfully isolated in C6/36 cells from DENV RNA positive blood samples. RT-PCR analysis of all supernatants and cells resulted negative even at 45 days after sample inoculation. To assess the possibility that virus present in the blood was in low quantity and therefore successful isolation was precluded, a quantitative RT-PCR was done. Even though the qRT-PCR was able to detect highly diluted DENV-1 through DENV-4 virus controls, the qRT-PCR of all positive samples showed to be under the detection limit (16.4 RNA copies per reaction) [30], which indicates therefore a probably low viremia in the sampled bats.

For detecting the presence of anti-dengue antibodies, a microneutralization test was performed with 241 bat sera samples with a final dilution of 1:20. We found an all-around DENV seroprevalence of 21.2% (51/241; Fig 1B, S3 Table). There were no differences in the seroprevalence found between males and females (χ^2^ = 0.35, df = 1, p = 0.55) nor juveniles and adults (χ^2^ = 0.03, df = 1, p = 0.86). We found antibodies against DENV in the species *B*. *plicata* (1/4), *G*. *soricina* (1/5), *M*. *pretiosus* (1/8), *M*. *rufus* (5/35), *M*. *sinaloae* (50/160), *R*. *io* (1/1), and *R*. *bickhami* (4/19). These bats belong to 4 different bat families which are strictly insectivorous, with the exception of *Glossophaga*, which is nectarivorous but also consumes fruit and insects [23]. Antibodies were present against DENV-1 in 25.5% (13/51), against DENV-2 in 23.5% (12/51), against DENV-3 in 70.6% (36/51), and against DENV-4 in 3.9% (2/51) of positive bats, with twelve individuals presenting antibodies against more than one serotype. Sarapiquí bats presented higher seroprevalence (χ^2^ = 7.31, df = 2, p = 0.03) with 30.67% (23/75), against DENV-1 (5/23), DENV-2 (7/23), and DENV-3 (18/23). Seroprevalence of Nicoya bats was lower at 17.1% (14/82), with the presence of antibodies against all four serotypes, DENV-1 (5/14), DENV-2 (1/14), DENV-3 (9/14), and DENV-4 (2/14). DENV seroprevalence of Central Valley bats was slightly lower at 16.67% (14/84), presenting antibodies against DENV-1 (3/14), DENV-2 (4/14), and DENV-3 (9/14). Interestingly, six individuals presented antibodies and DENV RNA in blood simultaneously, with 3 bats presenting antibodies against the same serotype detected in their blood that may suggest a putative incipient infection, and 3 bats with antibodies against a different serotype showing probably a potential secondary infection. Taking together, different rates of DENV seroprevalence in bats are observed in the three distinct locations sampled in Costa Rica.

### Captured mosquitoes PCR analyses show low dengue positivity and absence of bat blood feeding

To collect mosquitoes for dengue analyses and to determine if they were able to feed on bats, we set EVS CO_2_ traps in each sampled for bats household. We captured 651 mosquitoes, 121 males and 531 females from the following species: *Culex quinquefasciatus* (202), *C*. *nigripalpus* (315), *C*. *mollis* (1), *C*. *lactator* (16), *Culex* sp. (29), *Aedes aegypti* (54), *Ae*. *albopictus* (4), *Aedes* sp. (21), *Trichoprosopon digitatum* (2), *Limatus durhamii* (1), *Anopheles apicimacula* (2), *An*. *neivai* (1), and *Anopheles* sp. (3). *Aedes* mosquitoes were present in all sampling sites, though *Ae*. *albopictus* was only found in Sarapiquí, confirming its already reported presence in that particular area [36,37]. Most *Ae*. *aegypti* were collected in Nicoya during the rainy season, with just two exemplars captured in the Central Valley. Though measuring mosquito abundance was not our goal, no difference in abundance between sites was observed (Kruskal-Wallis, χ^2^ = 3.99, df = 2, p = 0.14). As expected but not statistically significant (Kruskal-Wallis, χ^2^ = 3.99, df = 2, p = 0.14), more mosquitoes were collected during rainy season, with the exception of the Central Valley where more mosquitoes were collected during the dry season. We pooled males (39 pools) and females (108 pools) corresponding to household and species sampled, hence number of mosquitoes in each pool differed. All male pools were negative for DENV RNA. Female pools were subdivided into heads and bodies for PCR analyses. DENV RNA presence in female mosquitoes was scarce. DENV-1 was detected only in one *Ae*. *aegypti* (1 head) and DENV-2 in a *Culex* sp. (1 body), both collected from the same household located in the Central Valley (low dengue incidence in humans). Also, DENV-3 was detected in a head pool of *Culex* sp. from Sarapiquí. All female body pools were tested for mosquito blood meal preference by detection of COI and *Cyt b*. After sequencing and blasting analyses, we detected human, dog, cat, rooster, horse, cattle, and rat blood, but no bat blood was found, suggesting that at least for the collected mosquitoes, bats that roost in houses with a given human proximity are not the main or even a source of feeding compared to other taxa.

### DENV seroprevalence of humans cohabiting with bats mirrors officially reported incidence in each area

No person surveyed presented any dengue symptomatic infection at sampling time. As expected, Nicoya presented higher seroprevalence in humans with a positivity of 82.6% (19/23). In Sarapiquí the seroprevalence was 16.7% (4/24) and in the Central Valley was 8.3% (1/12). Our results go in agreement with the epidemiological data published by the Ministry of Health, where in 2013 the incidence rate (number of new cases per 1000 persons at risk in a year) was 6592.1 in Nicoya, 2236.6 in Sarapiquí, and 2023.6 in the Central Valley (in the districts where we sampled). Whereas in 2014, the incidence was 677.4 in Nicoya, 773.8 in Sarapiquí, and 134 in the Central Valley [26] (S1 Fig). Therefore, high and low dengue incidence sampling site classification was performed appropriately. However, we found no correlation between human anti-dengue seroprevalence and bat dengue positivity among the sampled houses (GLMM Binomial, Z = -0.964, P>0.05, N = 235).

### Phylogenetic analyses suggest a spillover event from humans to bats

**Fig 2:**
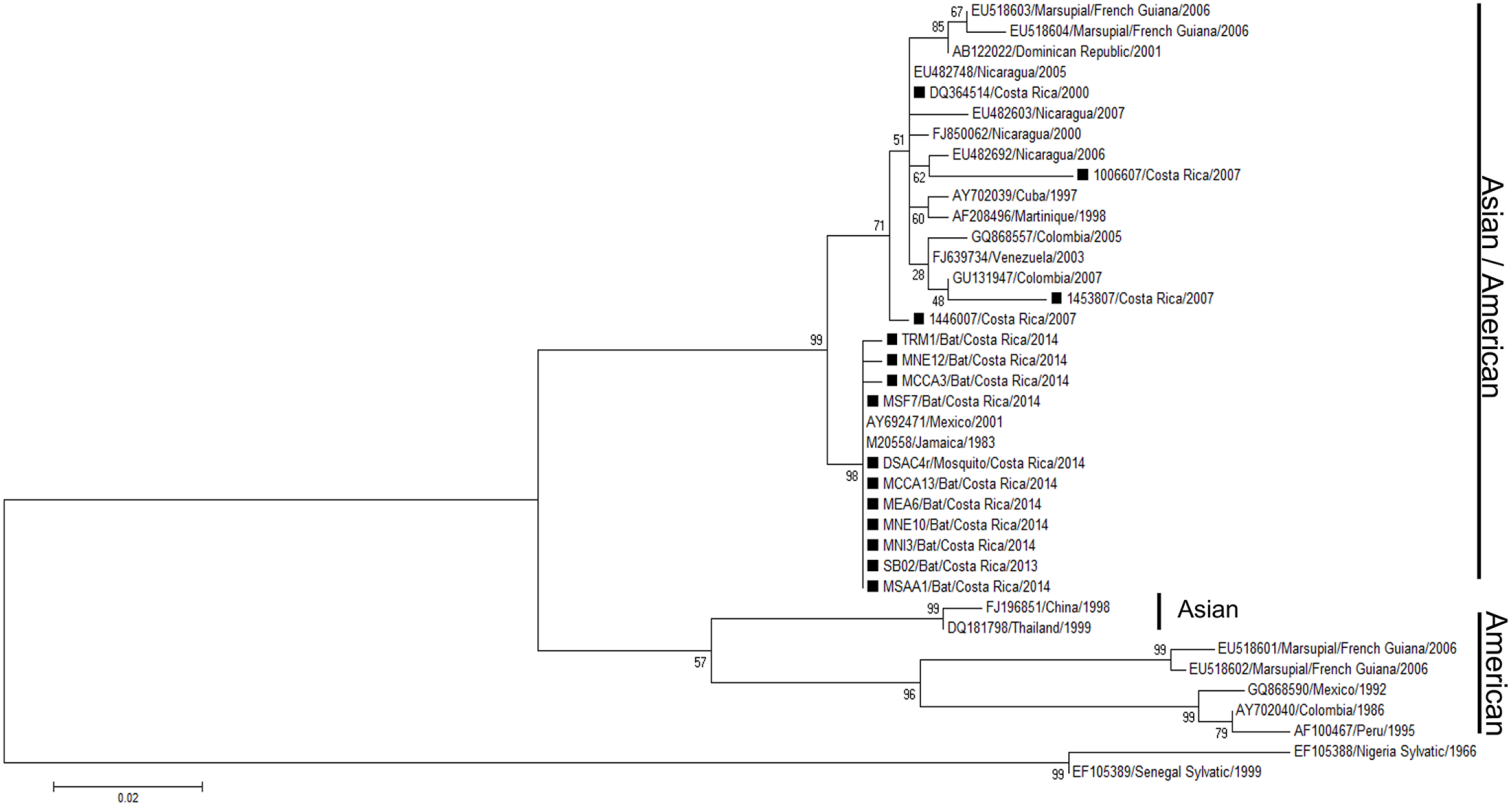
Maximum likelihood tree of 38 DENV-2 C/prM gene sequences (390 bp) including 10 from bats, 1 from a mosquito, and 3 from humans from former outbreaks in Costa Rica. Black rectangles (■) indicate the strains from Costa Rica. Bootstrap values are indicated at the respecting nodes. The sequences were named according to reference number/country/year of collection or detection.

**Fig 3:**
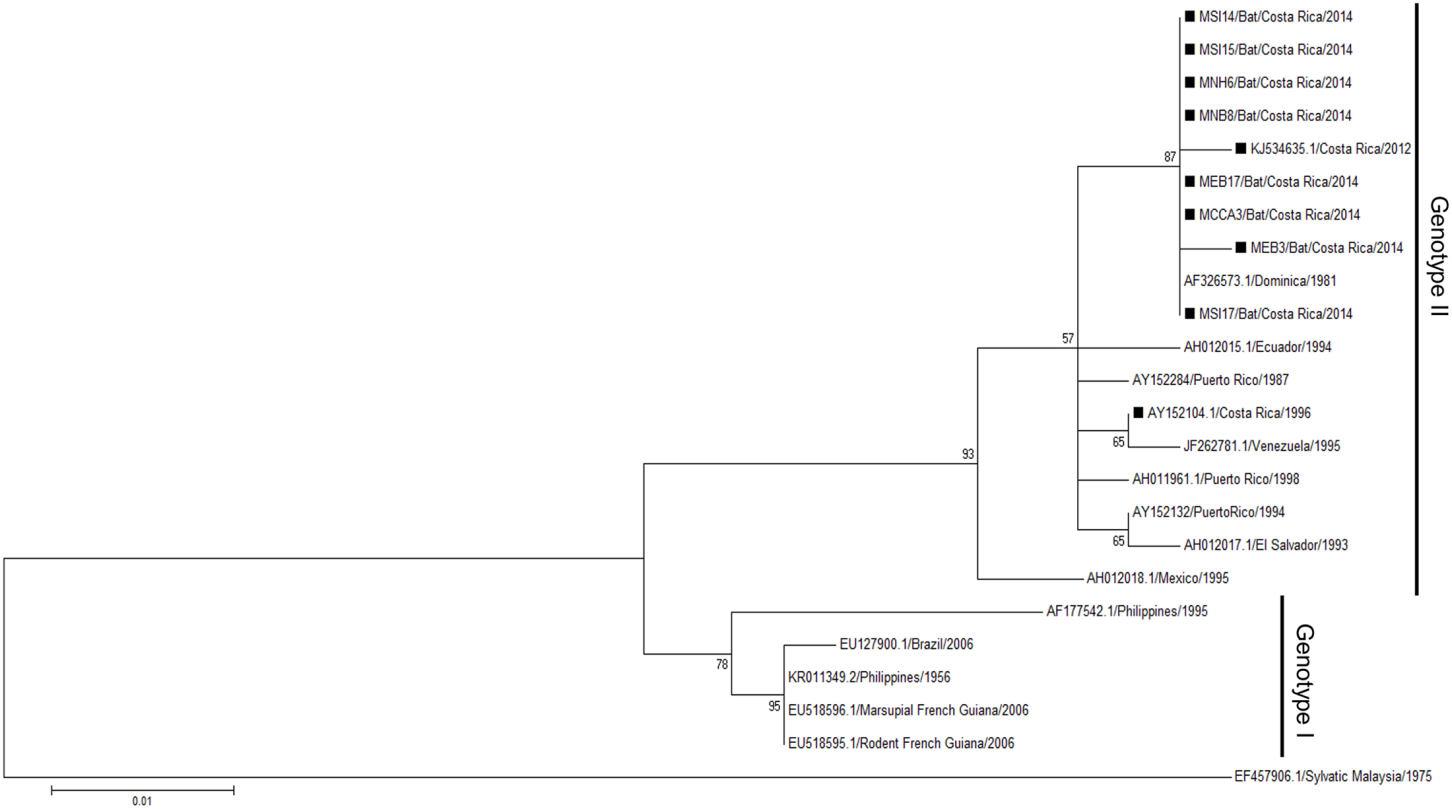
Maximum likelihood tree of 24 DENV-4 C/prM gene sequences (390 bp) including 8 from bats. Black rectangles (■) indicate the strains from Costa Rica. Bootstrap values are indicated at the respecting nodes. The sequences were named according to reference number/country/year of collection or detection.

In order to compare if viruses detected in bats, mosquitoes, and humans were similar, a phylogenetic analysis was performed. The short fragment obtained from the Díaz et al. screening PCR [33] is not suitable for genotyping, therefore we used a genotyping method described by de Thoisy et al. [5] and were able to obtain only C/prM sequences from DENV-2 (10 from bats and one from mosquito) and from DENV-4 (8 from bats). Phylogenetic trees were assembled using the maximum likelihood (ML) statistical method based on K2 + I for DENV-2 and K2 for DENV-4. A total of 38 DENV-2 sequences were used for this analysis: 24 from GenBank, 3 from a past outbreak [34] and 11 new sequences from this study (Fig 2; S4 Table). The retrieved sequences cluster together in the Asian/American genotype, the same reported genotype of strains from Nicaragua and Costa Rica. A total of 24 C/prM sequences of DENV-4 were included in the phylogenetic analysis: 16 from GenBank and 8 from bats (Fig 3; S5 Table). The sequences of bat cluster together in Genotype II, along with other sequences of DENV-4 reported from Costa Rica where a mosquito sequence was included (KJ534635.1, [37]). For both DENV-2 and DENV-4, we did not find any of the retrieved sequences to cluster together with sylvatic strains previously annotated in GenBank, but they all coincide with the genotypes of DENV currently circulating in humans.

## Discussion

Dengue is mainly maintained in large tropical urban areas via human-mosquito transmission cycles that no longer depend on animal reservoirs, though some reservoirs (non-human primates) are found in the jungles of Africa and Southeast Asia [38–40]. Recent studies have shown evidence of DENV presence in wildlife from the Neotropics, including bats [4–10]. But it is still unclear whether bats can maintain the virus and serve as reservoirs. Our results show that bats that cohabit with humans are critically exposed to DENV, since almost 9% sampled presented DENV RNA in blood and 22% had anti-DENV neutralizing antibodies. However, our findings suggest that bats are accidentally infected by DENV since: i) Although some studies have found the presence of NS1 in blood, hence virus replication [7], we were not able to detect virus RNA in any putative virus replication organ (heart, lung, kidney, liver, spleen and brain, though skin cells [41,42] or adipose tissue [43] were not analyzed); ii) Quantification of viral RNA by qRT-PCR in blood showed low RNA concentrations and these genome copies may not even be enclosed in intact or infective viral particles. This low concentration is found under the minimal mosquito infectious dose (MID) required to maintain the virus transmission cycle [44]. Moreover, the low concentration of virus RNA in blood precluded amplification of the 2.5 kbp region encompassing the prM/E genes [33]. Thus we were able only to obtain short length sequences (424–461 bp) from the C/prM region with a seminested PCR for sequencing and phylogenetic analyses [5]. Finally, iii) even if virus isolation is not always successful, we failed in attempts to isolate the virus from positive blood samples, suggesting no intact or infectious virus present. Taken together, under the current conditions of this study our results suggest that bats in these environments (sampled households) do not show sufficient virus replication, excluding them as potential hosts or reservoirs with no role in the transmission cycle, and making them feasible dead-end hosts for the virus. This confirms results obtained from previous independent studies where bats infected with DENV in controlled laboratory conditions failed to show viral amplification [11,12].

DENV RNA detected from the positive individuals corresponded to the DENV four serotypes. While DENV-1, -2 and -3 were causing dengue outbreaks in Costa Rica during 2013–2015, DENV-4 has not been reported in human samples from Costa Rica since 2002 [26]. Furthermore, in the human sera tested for this study and other serological studies performed in our lab, no human sera has shown monotypic anti-DENV-4 neutralizing response. However, DENV-4 was detected in an *Ae*. *albopictus* collected during 2015 from a pineapple field in Sarapiquí [37]. Besides competing with the other three serotypes, DENV-4 is the least frequently isolated serotype, it has not been associated with severe dengue outbreaks, and causes most of the clinically mild cases after dengue infection in humans [45,46]. Thus, DENV-4 may circulate unnoticed without its detection in health care facilities, and bats may function as sentinels showing exposure to this serotype.

We predicted to find more DENV positive bats in high human dengue incidence locations, yet we found positive bats in all sites sampled [26]. Also, we did not find any correlation between the DENV seroprevalence in humans sampled in the timespan this research was done and the presence of DENV positivity in bats. We also expected to find more positive individuals during the rainy season since the mosquito population augments and human cases increase considerably [26,47], but surprisingly in the Central Valley (low human incidence location) we found positive bats during both seasons. Furthermore, despite that we did not find a significant difference in the quantity of mosquitoes between seasons; as expected we collected more mosquitoes overall during the rainy season. Though, interestingly in the Central Valley, more mosquitoes were collected during the dry season. The presence of vector mosquitoes sustaining the viral transmission during the dry season has been associated with human habits such as saving water for the drought in artificial containers without proper management, presence of other type of containers such as flower vases, used tires, garbage and rubbish, therefore becoming all potential breeding sites [48]. The hyper endemic circulation of dengue and the presence of the mosquito vector in sampled sites may indicate enough potential sources for bats dengue exposure. Furthermore, the high seroprevalence observed in bats suggests a high exposure and rate of contact between bats and DENV. We detected more antibodies against DENV-3 (67.9%) and most of the bat sera studied showed a relative maturation in their neutralization response. Differences between age and gender have been reported in the immunological response against other viruses [49]. We found no significant difference, suggesting that males and females, adults and juveniles, are equally exposed to DENV. We did find higher bat seroprevalence in Sarapiquí compared to the other two sites. Sarapiquí has a greater rainfall amount yearly with a less drastic dry season, in comparison to Nicoya where human incidence is higher. This amount of rainfall nourishes populations of mosquitoes which may sustain viral transmission throughout the entire year. In six individuals we found concomitantly DENV RNA in blood and antibodies against DENV. Half of the individuals presented antibodies against the same serotype detected in blood, suggesting a previous or parallel immunological response. The other three individuals presented antibodies against a distinct serotype suggesting a potential secondary infection. Studies indicate that even after a controlled infection with a virus, bats do not always produce antibodies [50,51]. This observation displays how complex the humoral immunological response is in bats, thus making serological results difficult to interpret. Also, we have to take into account that so far, at least 19 different flaviviruses have been associated with bats [10,13,49–61]. Therefore, although PRNT is the gold standard for the serological diagnosis of flavivirus infections, results interpretation must be made with caution, and simultaneous assessment against all endemic flaviviruses must be performed for comparison of end-point titers to assure specific anti-DENV neutralizing antibodies [62]. Recently, Cabrera-Romo *et al*. [63] explored the role of bats as part of putative DENV sylvatic cycles in Mexico. They collected more than 200 bats of 18 different species from contrasting ecological settings with concurrent human DENV activity. RNA extracted from liver or spleen failed to show evidence for the presence of DENV nucleic acids, in agreement with our results. Nevertheless, their PRNT analyses showed no evidence of neutralizing anti-DENV antibodies. These contrasting results may be due to i) presence of anti-DENV neutralizing antibodies as a result of cross-reaction against other bat specific or human flaviviruses [62] as explained above; or ii) disparities in the bats species collected. The most frequent bat species collected by us belongs to the molossids, bat species absent from the Mexican study. In another of our recent publications [64], after primary embryonic cell culture of three different bat species, we observed a limited serotype-, organ-, and bat species- specific dengue susceptibility. Only some *Molossus-* but not *Artibeus* or *Desmodus*-derived primary cells sustained solely and poorly initial DENV-1 replication, though it was latter absent. These results confirm our current observations in molossids and reinforce the Cabrera-Romo *et al*. [63] findings; but noteworthy denote the importance on which bat species and which dengue serotype should be taken in account for further (if any) studies.

We hypothesized that bat infection will occur through an infected mosquito bite, but no mosquito was found positive for bat cytochrome b. However, our findings surmise that infection may occur through the oral ingestion of an infected mosquito. Studies have shown that bat infection is plausible after ingestion of mosquitoes infected with flaviviruses such as Yellow Fever Virus (YFV) and WNV [20,50,65]. Additionally, albeit we could not detect the virus in any putative replication organ, we detected two intestines from *M*. *sinaloae* positive for the same DENV serotype as found in their respective blood. Although this result may be caused by the presence of a positive dengue mosquito in the intestine lumen, it is tempting to speculate that some limited local viral replication in the bat intestine endothelial cells may be occurring. Supporting this conjecture, failure in recent studies attempting DENV infection of bats through a mosquito bite or virus inoculation may support an oral infection route [11,12]. Accordingly, the collected mosquitoes feeding preference did not indicate presence of bat blood, even though the EVC-CO_2_ traps were placed in close proximity to the bats roosting area. Moreover, the majority of the bats sampled by us are insectivorous, and even though the preferential food source for the molossids and other bigger bats may not be mosquitoes, it is possible that they will feed on them due to increased abundance [22,66]. Likewise, a nectarivorous bat as *G*. *soricina* could accidentally feed on dengue positive male mosquitoes while functioning as pollinators from flowers. Although the *Aedes* mosquitoes are diurnal, other nocturnal mosquitoes as *Culex*. which show limited virus replication in the gut [67] may be also a feeding and infection source. As well, bats could have been exposed to the virus in a different environment far from the roosting household.

Nevertheless, even though bats seem to get infected with DENV, they do not amplify the virus to a considerable extent to be able to transmit it to a mosquito. It seems that the exposure of bats to DENV is accidental, becoming an example of spill over from humans to bats as reported by de Thoisy *et al*. [5] with samples of wildlife taken in close proximity to human settlements where dengue outbreaks ensue. This is supported not only by our results in the phylogenetic analysis, where the dengue strains sequenced from bats and mosquitoes cluster together in close relation with the reported strains of dengue in this and neighboring countries (Figs 2 and 3), but also by not showing any histopathological findings suggestive of infection in all analyzed tissues. Even if we cannot determine the route of contact occurring between the virus and the bat, our results suggest that bats are an epidemic dead end for this virus

Several viruses have been detected in bat tissues or excreta; however, this does not prove causation of disease [16]. Some of these viruses or viral sequences might have been acquired from food eaten by bats and could be irrelevant with respect to viral disease epidemiology. Many knowledge gaps connecting bats and zoonotic viruses exist, thus linking bats with these events without strong evidence is a disservice with negative consequences [13]. For example, investing efforts in controlling the wrong reservoir can postpone suitable mitigation actions that could prevent deaths or interrupt disease spread; and a potential ‘pest control’ of bat populations may deny us their important ecosystemic services [13]. Therefore as stated by Moratelli and Calisher, after understanding the role of bats (or wildlife) in the maintenance and circulation of pathogens and the mechanisms underlying the emergence of zoonotic diseases, wildlife biologists and epidemiologists should work together developing appropriate management plans to control virus circulation [13]. Just then, risks of human infection without causing significant biases against specific animal populations will be minimized.

## Supporting information

S1 Fig Sampling sites in Costa Rica and their dengue incidence during study years, 2013 and 2014. The black squares, diamonds and triangles (■, ♦, ▲) represent a sampled household. The incidence values were retrieved from epidemiological surveillance done by the Ministry of Health [26]. Map was created using QGIS 2.14.3 (http://www.qgis.org/en/site/) and DIVA GIS maps (http://www.diva-gis.org). Baselayer data was obtained from http://www.diva-gis.org/gdata.
(TIF)

S1 Table Primers used in the study. (DOCX)

S2 Table Prevalence of DENV RNA obtained by PCR from each serotype (D1-4) in all captured bat species in the 3 sites of study in Costa Rica (Nicoya, Sarapiquí and Central Valley) during the dry and rainy season, 2013–2014. (DOCX)

S3 Table Seroprevalence against each dengue serotype obtained from serum diluted 1:20 by PRNT_90_ from bats captured in the 3 sites of study in Costa Rica (Nicoya, Sarapiquí and Central Valley) during 2013–2014. (DOCX)

S4 Table Information of DENV-2 sequences used in phylogenetic analysis. (DOCX)

S5 Table Information of DENV-4 sequences used in phylogenetic analysis. (DOCX)
