# Supplementary figures and images for: Neotropical bats that co-habit with humans function as dead-end hosts for dengue virus
Source: PLoS Negl Trop Dis. 2017 May 18;11(5):e0005537. doi: 10.1371/journal.pntd.0005537 (PMC5451070; doi:10.1371/journal.pntd.0005537)

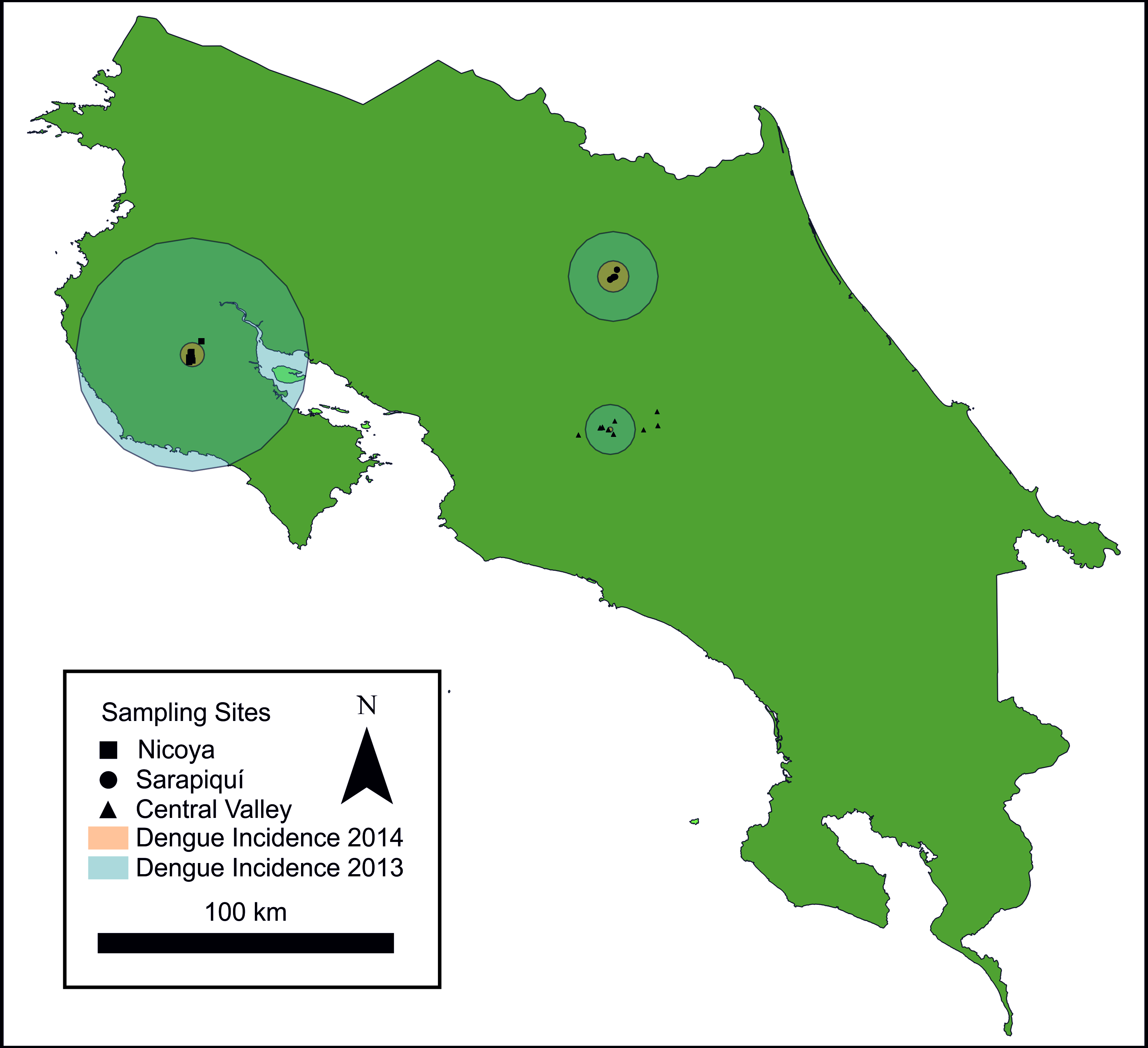

Supplement: S1 Fig — The black squares, diamonds and triangles (■, ♦, ▲) represent a sampled household. The incidence values were retrieved from epidemiological surveillance done by the Ministry of Health [26]. Map was created using QGIS 2.14.3 (http://www.qgis.org/en/site/) and DIVA GIS maps (http://www.diva-gis.org). Baselayer data was obtained from http://www.diva-gis.org/gdata. (TIF) [file pntd.0005537.s001.tif]
