# Supplementary material for: Neotropical bats that co-habit with humans function as dead-end hosts for dengue virus
Source: PLoS Negl Trop Dis. 2017 May 18;11(5):e0005537. doi: 10.1371/journal.pntd.0005537 (PMC5451070; doi:10.1371/journal.pntd.0005537)
Supplement: S1 Table — (DOCX) [file pntd.0005537.s002.docx]

Supplementary Table 1. Primers used in the study.

| Primer | Sequence 5’-3’ | Genome position | Use | Reference |
| --- | --- | --- | --- | --- |
| D1 | TCAATATGCTGAAACGCGCGAGAAACCG | 134-161 | PCR | Lanciotti *et al.* 1992 |
| D2 | TTGCACCAACAGTCAATGTCTTCAGGTTC | 616-644 | PCR | Lanciotti *et al.* 1992 |
| TS1 | CGTCTCAGTGATCCGGGGG | 568-586 | PCR | Lanciotti *et al.* 1992 |
| TS2 | CGCCACAAGGGCCATGAACAG | 232-252 | PCR | Lanciotti *et al.* 1992 |
| TS3 | TAACATCATCATGAGACAGAGC | 400-421 | PCR | Lanciotti *et al.* 1992 |
| TS4 | CTCTGTTGTCTTAAACAAGAGA | 506-527 | PCR | Lanciotti *et al.* 1992 |
| DenS | GGATAGACCAGAGATCCTGCTGT | 10615-10636 | qPCR | Drosten *et al.* 2002 |
| DenAs | CATTCCATTTTCTGGCGTTC plus CAATCCATCTTGCGGCGCTC | 10694-10675 | qPCR | Drosten *et al.* 2002 |
| DenP | CAGCATCATTCCAGGCACAG | 10656-10675 | qPCR probe | Drosten *et al.* 2002 |
| COI_long (f) | AACCACAAAGACATTGGCAC | 5934^a^ | PCR | Townzen *et al.* 2008 |
| COI_long (r) | AAGAATCAGAATARGTGTTG | 6597 ^a^ | PCR | Townzen *et al.* 2008 |
| COI_short (f) | GCAGGAACAGGWTGAACCG | 6267 ^a^ | PCR | Townzen *et al.* 2008 |
| COI_short (r) | AATCAGAAYAGGTGTTGGTATAG | 6591 ^a^ | PCR | Townzen *et al.* 2008 |
| Cyt *b* (f) | GAGGMCAAATATCATTCTGAGG | 15 150 ^a^ | PCR | Townzen *et al.* 2008 |
| Cyt *b* (r) | TAGGGCVAGGACTCCTCCTAGT | 15 607 ^a^ | PCR | Townzen *et al.* 2008 |
| D1 | TCAATATGCTGAAACGCGCGAGAAACCG | 134-161^b^ | PCR, Sequencing | Díaz *et al.* 2006 |
| D1-682F | AACCGGYGAACACCGACGAGA | 682 | Sequencing | Díaz *et al.* 2006 |
| D1-1064F | GAACTCTTGAAGACGGAGGTCACGAA | 1064 | Sequencing | Díaz *et al.* 2006 |
| D1-1167R | TTGTTCTTCCACCAGTGTAGCCTCTC | 1167 | Sequencing | Díaz *et al.* 2006 |
| D1-1488F | GCTCACCTAGAACAGGGCTGGACTTT | 1488 | Sequencing | Díaz *et al.* 2006 |
| D1-1649R | TTCTTTGCATGAGCTGTCTTGAATGT | 1649 | Sequencing | Díaz *et al.* 2006 |
| D1-1972F | CCAGAATGGGAGATTGATAACA | 1972 | Sequencing | Díaz *et al.* 2006 |
| D1-2125R | CGGTTGCTTCGAACATTTTCCCTATG | 2125 | Sequencing | Díaz *et al.* 2006 |
| D1-2726R | ATGGGTTGTGGCCTAATCAT | 2726 | PCR, Sequencing | Díaz *et al.* 2006 |
| D2-739F | ATGGGATTGGAGACACGAACTGAA | 739 | Sequencing | Díaz *et al.* 2006 |
| D2-1184F | ATGAAGAGCAGGACAAAAGGTT | 1184 | Sequencing | Díaz *et al.* 2006 |
| D2-1225R | CCATTTCCCCATCCTCTGTCTAC | 1225 | Sequencing | Díaz *et al.* 2006 |
| D2-1540F | GAAGACAAAGCTTGGCTGGTG | 1540 | Sequencing | Díaz *et al.* 2006 |
| D2-1648R | GCATGGGGATTTTTGAARGTGAC | 1648 | Sequencing | Díaz *et al.* 2006 |
| D2-2028F | GGAGGTTCTGCTTCTATGTTGACT | 2028 | Sequencing | Díaz *et al.* 2006 |
| D2-2120R | TCAAACATTTGGCCGATRGAACTTC | 2120 | Sequencing | Díaz *et al.* 2006 |
| D2-2588R | TCTTGTTACTGAGCGGATTC | 2588 | PCR, Sequencing | Díaz *et al.* 2006 |
| D3-644F | CTTACATCAACATGGGTGACTTAT | 644 | Sequencing | Díaz *et al.* 2006 |
| D3-1343F | TACACCGTCATCATCACAGTG | 1343 | Sequencing | Díaz *et al.* 2006 |
| D3-1390R | CTTACATCAACATGGGTGACTTAT | 1390 | Sequencing | Díaz *et al.* 2006 |
| D3-1671F | AAGAAGTAGTTGTCCTTGGAT | 1671 | Sequencing | Díaz *et al.* 2006 |
| D3-1953R | CATTGTGAGCTTTCCCTTGTC | 1953 | Sequencing | Díaz *et al.* 2006 |
| D3-2429R | TTCTTTGCCTTTCCAGTTTAT RT | 2429 | PCR, Sequencing | Díaz *et al.* 2006 |
| D4-762F | GAGACATGGATGTCATCGGAAGG | 762 | Sequencing | Díaz *et al.* 2006 |
| D4-1240F | GGGGCAATGGCTGTGGCTTGTT | 1240 | Sequencing | Díaz *et al.* 2006 |
| D4-1308R | TTGGACCAAATTGCCTGTTATCTT | 1308 | Sequencing | Díaz *et al.* 2006 |
| D4-1667F | YCATGCCAAGAGACAGGATGTGAC | 1667 | Sequencing | Díaz *et al.* 2006 |
| D4-1702R | GCAAGAATGCATGGCTCCTTCCTGAG | 1702 | Sequencing | Díaz *et al.* 2006 |
| D4-2158R | GAATGGCCATTCGTTTTGCACCTC | 2158 | Sequencing | Díaz *et al.* 2006 |
| D4-2052F | CCYTTTGGGGACAGCGCTACATA | 2052 | Sequencing | Díaz *et al.* 2006 |
| D4-2649R | TGTCCTCCTTCCCAGAGAACATAGTT | 2649 | PCR, Sequencing | Díaz *et al.* 2006 |
| D1L | TAGGTCATTGTGTCCTCACATAACTCTCC | 560-588 | PCR, sequencing | De Thoisy *et al.* 2009 |
| D2L | CTTGTACGTGATTGTATCTTCACACA | 569-594 | PCR, sequencing | De Thoisy *et al.* 2009 |
| D3L | TTGTAAGTGACCGTGTCATCACACAT | 566-594 | PCR, sequencing | De Thoisy *et al.* 2009 |
| D4L | TCCATGGCAATGAGAGTGCATTTGTTGA | 533-560 | PCR, sequencing | De Thoisy *et al.* 2009 |

^a^Location on human mtDNA

^b^The priming position of primer D1 in each genome was as follows: type 1: 132; type 2: 134; type 3: 132; type 4: 137.
