# Supplementary material for: Neotropical bats that co-habit with humans function as dead-end hosts for dengue virus
Source: PLoS Negl Trop Dis. 2017 May 18;11(5):e0005537. doi: 10.1371/journal.pntd.0005537 (PMC5451070; doi:10.1371/journal.pntd.0005537)
Supplement: S2 Table — (DOCX) [file pntd.0005537.s003.docx]

Supplementary Table 2. Prevalence of DENV RNA obtained by PCR from each serotype (D1-4) in each species of bats captured in the 3 sites of study in Costa Rica (Nicoya, Sarapiquí and Central Valley) during the dry and rainy season, 2013 -2014.

|  | Nicoya (N) | | | | N Total | Sarapiquí (S) | | | | | | S Total | Central Valley (CV) | | | | | | | CV Total | Total |
| --- | --- | --- | --- | --- | --- | --- | --- | --- | --- | --- | --- | --- | --- | --- | --- | --- | --- | --- | --- | --- | --- |
| Species | Dry | Rainy | | |  | Dry | Rainy | | | | |  | Dry | | | Rainy | | | |  |  |
|  | n | n | D2 | D4 |  | n | n | D2 | D3 | | D4 |  | n | D1 | D2 | n | D1 | D2 | D4 |  |  |
| *Balantiopteryx plicata* |  | 5 |  |  | 5 |  |  |  |  |  | |  |  |  |  |  |  |  |  |  | 5 |
| *Eptesicus fuscus* |  |  |  |  |  |  |  |  |  |  | |  |  |  |  | 3 |  |  |  | 3 | 3 |
| *Eumops glaucinus* |  |  |  |  |  |  |  |  |  |  | |  |  |  |  | 3 | 1 |  |  | 3 | 3 |
| *Glossophaga soricina* |  |  |  |  |  |  |  |  |  |  | |  | 10 |  | 1 |  |  |  |  | 10 | 10 |
| *Molossus pretiosus* |  | 10 | 1 |  | 10 |  |  |  |  |  | |  |  |  |  |  |  |  |  |  | 10 |
| *Molossus rufus* |  |  |  |  |  |  |  |  |  |  | |  | 24 |  | 2 | 30 |  | 2** | 1** | 54 | 54 |
| *Molossus sinaloae* | 40 | 42 | 2 | 3 | 82 | 35 | 62 | 3* | 2* | 3 | | 97 | 10 |  | 2 | 18 |  |  | 2 | 28 | 207 |
| *Myotis elegans* |  |  |  |  |  |  | 1 |  |  |  | | 1 |  |  |  |  |  |  |  |  | 1 |
| *Myotis nigricans* |  |  |  |  |  | 2 | 1 |  |  |  | | 3 |  |  |  |  |  |  |  |  | 3 |
| *Rhogeessa io* |  |  |  |  |  |  | 1 |  |  |  | | 1 |  |  |  |  |  |  |  |  | 1 |
| *Rhogeessa bickami* |  |  |  |  |  |  |  |  |  |  | |  | 10 | 1 | 1 | 10 | 3 |  | 1 | 20 | 20 |
| *Uroderma convexum* | 1 |  |  |  | 1 |  |  |  |  |  | |  |  |  |  |  |  |  |  |  | 1 |
| Total | 41 | 57 | 3 | 3 | 98 | 37 | 65 | 3 | 2 | 3 | | 102 | 54 | 1 | 6 | 64 | 4 | 2 | 4 | 118 | 318 |

*Two individuals with joint detection of DENV-2 and DENV-4. **One individual with joint detection of DENV-2 and DENV-3
