# Supplementary material for: Neotropical bats that co-habit with humans function as dead-end hosts for dengue virus
Source: PLoS Negl Trop Dis. 2017 May 18;11(5):e0005537. doi: 10.1371/journal.pntd.0005537 (PMC5451070; doi:10.1371/journal.pntd.0005537)
Supplement: S3 Table — (DOCX) [file pntd.0005537.s004.docx]

Supplementary Table 3. Seroprevalence against each dengue serotype obtained from serum diluted 1:20 by PRNT90 from bats captured in the 3 sites of study in Costa Rica (Nicoya, Sarapiquí and Central Valley) during the dry and rainy season, 2013 -2014.

| Species | Nicoya (N) | | | | N Total | Sarapiquí (S) | | | S Total | Central Valley (CV) | | | CV Total | Total |
| --- | --- | --- | --- | --- | --- | --- | --- | --- | --- | --- | --- | --- | --- | --- |
|  | D1 | D2 | D3 | D4 |  | D1 | D2 | D3 |  | D1 | D2 | D3 |  |  |
| *Balantiopteryx plicata* |  |  | 1 |  | 4 |  |  |  |  |  |  |  |  | 4 |
| *Eptesicus fuscus* |  |  |  |  |  |  |  |  |  |  |  |  | 3 | 3 |
| *Eumops glaucinus* |  |  |  |  |  |  |  |  |  |  |  |  | 3 | 3 |
| *Glossophaga soricina* |  |  |  |  |  |  |  |  |  |  |  | 1 | 5 | 5 |
| *Molossus pretiosus* |  | 1 |  |  | 8 |  |  |  |  |  |  |  |  | 8 |
| *Molossus rufus* |  |  |  |  |  |  |  |  |  | 2 | 1 | 2 | 35 | 35 |
| *Molossus sinaloae* | 5 |  | 8 | 2 | 69 | 5 | 7 | 17 | 72 | 1 |  | 5 | 19 | 160 |
| *Myotis elegans* |  |  |  |  |  |  |  |  | 1 |  |  |  |  | 1 |
| *Myotis nigricans* |  |  |  |  |  |  |  |  | 1 |  |  |  |  | 1 |
| *Rhogeessa io* |  |  |  |  |  |  |  | 1 | 1 |  |  |  |  | 1 |
| *Rhogeessa bickami* |  |  |  |  |  |  |  |  |  | 2 | 2 |  | 19 | 19 |
| *Uroderma convexum* |  |  |  |  | 1 |  |  |  |  |  |  |  |  | 1 |
| Total | 5 | 1 | 9 | 2 | 82 | 5 | 7 | 18 | 75 | 5 | 3 | 8 | 84 | 241 |
