# Supplementary material for: Neotropical bats that co-habit with humans function as dead-end hosts for dengue virus
Source: PLoS Negl Trop Dis. 2017 May 18;11(5):e0005537. doi: 10.1371/journal.pntd.0005537 (PMC5451070; doi:10.1371/journal.pntd.0005537)
Supplement: S4 Table — (DOCX) [file pntd.0005537.s005.docx]

**Supplementary Table 4.** Information of DENV-2 sequences used in phylogenetic analysis.

| **DENVs strains in this study** | **GenBank Accession number** | **Country** | **Year** | **Strain** | **Genotype** | |
| --- | --- | --- | --- | --- | --- | --- |
| AY702040/Colombia/ 1986 | AY702040 | Colombia | 1986 | DENV-2/CO/I348600/1986 | American | |
| AF100467/Peru/1995 | AF100467 | Peru | 1995 | DENV-2/PE/IQT1797/1995 | American | |
| GQ868590/Mexico/ 1992 | GQ868590 | Mexico | 1992 | DENV-2/MX/BID-V3356/1992 | American | |
| EU518601/Marsupial/ French Guiana/2006 | EU518601 | French Guiana | 2006 | DENV-2/FG/B1032/2006 | American | |
| EU518602/Marsupial/ French Guiana/2006 | EU518602 | French Guiana | 2006 | DENV-2/FG/B1015/2006 | American | |
| EU518603/Marsupial/ French Guiana/2006 | EU518603 | French Guiana | 2006 | DENV-2/FG/B1010/2006 | Asian/ American | |
| EU518604/Marsupial/ French Guiana/2006 | EU518604 | French Guiana | 2006 | DENV-2/FG/B1004/2006 | Asian/ American | |
| EU482748/Nicaragua/ 2005 | EU482748 | Nicaragua | 2005 | DENV-2/NI/BID-V513/2005 | Asian/ American | |
| EU482603/Nicaragua/ 2007 | EU482603 | Nicaragua | 2007 | DENV-2/NI/BID-V615/2007 | Asian/ American | |
| EU482692/Nicaragua/ 2006 | EU482692 | Nicaragua | 2006 | DENV-2/NI/BID-V580/2006 | Asian/ American | |
| FJ850062/Nicaragua/ 2000 | FJ850062 | Nicaragua | 2000 | DENV-2/NI/BID-V2659/2000 | Asian/ American | |
| FJ639734/Venezuela/ 2003 | FJ639734 | Venezuela | 2003 | DENV-2/VE/BID-V2160/2003 | Asian/ American | |
| GQ868557/Colombia/ 2005 | GQ868557 | Colombia | 2005 | DENV-2/CO/BID-V3373/2005 | Asian/ American | |
| GU131947/Colombia/ 2007 | GU131947 | Colombia | 2007 | DENV-2/CO/BID-V3374/2007 | Asian/ American | |
| DQ364514/Costa Rica/2000 | DQ364514 | Costa Rica | 2000 | DENV-2/CR/CR_7945_00/2000 | Asian/ American | |
| AY702039/Cuba/ 1997 | AY702039 | Cuba | 1997 | DENV-2/CU/Cuba205_97/1997 | Asian/ American | |
| AF208496/Martinique/ 1998 | AF208496 | Martinique | 1998 | DENV-2/MTQ/H_IMTSSA_MART_98-703/1998 | Asian/ American | |
| AB122022/Dominican Republic/2001 | AB122022 | Dominican Republic | 2001 | DENV-2/DOM/DR59_01/2001 | Asian/ American | |
| AY692471/Mexico/ 2001 | AY692471 | Mexico | 2001 | DENV-2/MX/HUAT12/2001 | Asian/ American | |
| M20558/Jamaica/ 1983 | M20558 | Jamaica | 1983 | DENV-2/JAM/Jamaica_N.1409/1983 | Asian/ American | |
| DQ181798/Thailand/ 1999 | DQ181798 | Thailand | 1999 | DENV-2/TH/ThD2_0055_99/1999 | Asian | |
| FJ196851/China/1998 | FJ196851 | China | 1998 | DENV-2/CH/GD08_98/1998 | Asian | |
| EF105389/Senegal Sylvatic/1999 | EF105389 | Senegal | 1999 | DENV-2/SN/Dak Ar 141069/1999 | Sylvatic | |
| EF105388/Nigeria Sylvatic/1966 | EF105388 | Nigeria | 1966 | DENV-2/NG/IBH11664/1966 | Sylvatic | |
| 1006607/Costa Rica/2007 | KY461756 | Costa Rica | 2007 | DENV-2/CR/10066-07/2007 | Asian/ American | |
| 1446007/Costa Rica/2007 | KY461758 | Costa Rica | 2007 | DENV-2/CR/14460-07/2007 | Asian/ American | |
| 1353807/Costa Rica/2007 | KY461757 | Costa Rica | 2007 | DENV-2/CR/13538-07/2007 | Asian/ American | |
| DSAC4r/**Mosquito**/Costa Rica/2014 | KY461759 | Costa Rica | 2014 | DENV-2/CR/DSAC4r/2014 | Asian/ American |  |
| MCCA3/**Bat**/Costa Rica/2014 | KY461760 | Costa Rica | 2014 | DENV-2/CR/MCCA3/2014 | Asian/ American |  |
| MCCA13/**Bat**/Costa Rica/2014 | KY461761 | Costa Rica | 2014 | DENV-2/CR/MCCA13/2014 | Asian/ American |  |
| MEA6/**Bat**/Costa Rica/2014 | KY461762 | Costa Rica | 2014 | DENV-2/CR/MEA6/2014 | Asian/ American | |
| MNE10/**Bat**/Costa Rica/2014 | KY461763 | Costa Rica | 2014 | DENV-2/CR/MNE10/2014 | Asian/ American |  |
| MNE12/**Bat**/Costa Rica/2014 | KY461764 | Costa Rica | 2014 | DENV-2/CR/MNE12/2014 | Asian/ American |  |
| MNI3/**Bat**/Costa Rica/2014 | KY461765 | Costa Rica | 2014 | DENV-2/CR/MNI3/2014 | Asian/ American | |
| MSAA1/**Bat**/Costa Rica/2014 | KY461766 | Costa Rica | 2014 | DENV-2/CR/MSAA1/2014 | Asian/ American |  |
| MSF7/**Bat**/Costa Rica/2014 | KY461767 | Costa Rica | 2014 | DENV-2/CR/MSF7/2014 | Asian/ American | |
| SB02/**Bat**/Costa Rica/2013 | KY461768 | Costa Rica | 2013 | DENV-2/CR/SB02/2013 | Asian/ American | |
| TRM1/**Bat**/Costa Rica/2014 | KY461769 | Costa Rica | 2014 | DENV-2/CR/TRM1/2014 | Asian/ American | |
