# Supplementary material for: Neotropical bats that co-habit with humans function as dead-end hosts for dengue virus
Source: PLoS Negl Trop Dis. 2017 May 18;11(5):e0005537. doi: 10.1371/journal.pntd.0005537 (PMC5451070; doi:10.1371/journal.pntd.0005537)
Supplement: S5 Table — (DOCX) [file pntd.0005537.s006.docx]

| **DENVs strains in this study** | **GenBank Accession number** | **Country** | **Year** | **Strain** | **Genotype** |
| --- | --- | --- | --- | --- | --- |
| KR011349.2/Philippines/1956 | KR011349.2 | Philippines | 1956 | DENV-4/PH/H241/1956 | Genotype I |
| AF177542.1/Philippines/1995 | AF177542.1 | Philippines | 1995 | DENV-4/PH/ Mindanao_BDJ_1995/1995 | Genotype I |
| EU518596.1/Marsupial French Guiana/2006 | EU518596.1 | French Guiana | 2006 | DENV-4/FG/B1008/2006 | Genotype I |
| EU518595.1/Rodent French Guiana/2006 | EU518595.1 | French Guiana | 2006 | DENV-4/FG/B1106/2006 | Genotype I |
| AF326573.1/Dominica/ 1981 | AF326573.1 | Dominican Republic | 1981 | DENV-4/DOM/814669/1981 | Genotype II |
| AH012015.1/Ecuador/ 1994 | AH012015.1 | Ecuador | 1994 | DENV-4/EC/ D4.109_1994EC/1994 | Genotype II |
| AY152284/Puerto Rico/1987 | AY152284 | Puerto Rico | 1987 | DENV-4/PR/D4.9_1987/1987 | Genotype II |
| AH011961.1/Puerto Rico/1998 | AH011961.1 | Puerto Rico | 1998 | DENV-4/PR/D4.48_1998/1998 | Genotype II |
| JF262781.1/Venezuela/ 1995 | JF262781.1 | Venezuela | 1995 | DENV-4/VE/INH6412/1995 | Genotype II |
| EU127900.1/Brazil/2006 | EU127900.1 | Brazil | 2006 | DENV-4/BR/AM1619/2006 | Genotype II |
| AY152132/PuertoRico/ 1994 | AY152132 | PuertoRico | 1994 | DENV-4/PR/D4.77_1994/1994 | Genotype II |
| AH012018.1/Mexico/ 1995 | AH012018.1 | Mexico | 1995 | DENV-4/MX/D4.111_1995MX/1995 | Genotype II |
| KJ534635.1/Costa Rica/2012 | KJ534635.1 | Costa Rica | 2012 | DENV-4/CR/P30/2012 | Genotype II |
| AY152104.1/Costa Rica/1996 | AY152104.1 | Costa Rica | 1996 | DENV-4/CR/D4.108_1996CR/1996 | Genotype II |
| AH012017.1/El Salvador/1993 | AH012017.1 | El Salvador | 1993 | DENV-4/ES/ D4.110_1993ES/1993 | Genotype II |
| EF457906.1/Sylvatic Malaysia/1975 | EF457906.1 | Sylvatic Malaysia | 1975 | DENV-4/MY/P75-215/1975 | Sylvatic |
| MCCA3/Bat/Costa Rica/2014 | KY461770 | Costa Rica | 2014 | DENV-4/CR/MCCA3/2014 | Genotype II |
| MEB3/**Bat**/Costa Rica/2014 | KY461771 | Costa Rica | 2014 | DENV-4/CR/MEB3/2014 | Genotype II |
| MEB17/**Bat**/Costa Rica/2014 | KY461772 | Costa Rica | 2014 | DENV-4/CR/MEB17/2014 | Genotype II |
| MNB8/**Bat**/Costa Rica/2014 | KY474382 | Costa Rica | 2014 | DENV-4/CR/MNB8/2014 | Genotype II |
| MNH6/**Bat**/Costa Rica/2014 | KY461773 | Costa Rica | 2014 | DENV-4/CR/MNH6/2014 | Genotype II |
| MSI14/**Bat**/Costa Rica/2014 | KY461774 | Costa Rica | 2014 | DENV-4/CR/MSI14/2014 | Genotype II |
| MSI15/**Bat**/Costa Rica/2014 | KY461775 | Costa Rica | 2014 | DENV-4/CR/MSI15/2014 | Genotype II |
| MSI17/**Bat**/Costa Rica/2014 | KY461776 | Costa Rica | 2014 | DENV-4/CR/MSI17/2014 | Genotype II |
|  |  |  |  |  |  |

Supplementary Table 5. **Information of DENV-4 sequences used in phylogenetic analysis.**
